# Antimicrobial Activity of 4-Chlorocinnamic Acid Derivatives

**DOI:** 10.1155/2019/3941242

**Published:** 2019-04-23

**Authors:** Rayanne H. N. Silva, Ana C. M. Andrade, Diego F. Nóbrega, Ricardo D. de Castro, Hilzeth L. F. Pessôa, Nidhi Rani, Damião P. de Sousa

**Affiliations:** ^1^Laboratory of Pharmaceutical Chemistry, Department of Pharmaceutical Sciences, Federal University of Paraíba, Cidade Universitária, João Pessoa, Paraíba 58051-900, Brazil; ^2^Laboratory of Experimental Pharmacology and Cell Culture of the Health Sciences Center, Federal University of Paraíba, Cidade Universitária, João Pessoa, Paraíba 58051-900, Brazil; ^3^Labetox, Research Institute in Pharmacons and Medicines, Federal University of Paraíba, Cidade Universitária, João Pessoa, Paraíba 58051-900, Brazil; ^4^Maharishi Markandeshwar School of Pharmacy, Maharishi Markandeshwar University, Sadopur, Ambala 134007, Haryana, India

## Abstract

The microbial resistance of fungi and bacteria is currently considered a major public health problem. Esters derived from cinnamic acid have a broad spectrum of pharmacological properties that include antimicrobial activity. In this study, a collection of structurally related 4-chlorocinnamic acid esters was prepared using Fischer esterification reactions, alkyl or aryl halide esterification, and Mitsunobu and Steglich reactions. All of the esters were submitted to antimicrobial tests against strains of the species* Candida albicans, Candida glabrata, Candida krusei, Candida guilliermondii, Pseudomonas aeruginosa*, and* Staphylococcus aureus*. The compounds also were subjected to molecular docking study with the enzyme 14*α*-demethylase. Twelve esters derived from 4-chlorocinnamic acid were obtained, with yields varying from 26.3% to 97.6%, three of which were unpublished. The ester methyl 4-chlorocinnamate (**1**) presented activity against* S. aureus* at the highest concentration tested. In the antifungal evaluation, all of the esters were bioactive, but methoxyethyl 4-chlorocinnamate (**4**) and perillyl 4-chlorocinnamate (**11**) were the most potent (MIC = 0.13 and 0.024 *μ*mol/mL, respectively). The data of molecular docking suggested that all the compounds present good affinity towards the active site related to antifungal activity. Therefore, the esters tested may be inhibitors of the enzyme 14*α*-demethylase. In addition, the results demonstrate that substituents of short alkyl chains with presence of heteroatom, such as oxygen, or those with a perillyl type terpenic substructure promote better antifungal profiles.

## 1. Introduction

Infectious diseases caused by microorganisms such as bacteria and fungi remain a prominent global health problem, especially in developing and low-income countries [1, 2]. Bacteria are becoming highly drug resistant, creating a growing problem in medicine. Several types of resistant bacterial infections are ever more difficult to treat, increasing morbidity and mortality and causing high health care costs [3]. Similarly, fungal infections also pose a challenge to medical practice; significant increases in indicators of morbidity and mortality have been observed, especially in hospitalized immunocompromised individuals [4, 5].

Of the most common fungal infections, the most frequent etiological agent is* C. albicans*. However, other species of the* Candida* genus can also be included such as* C. guilliermondii, C. parapsilosis, C. stellatoidea, C. tropicalis, C. glabrata*, and* C. krusei*, which are recognized for resistance to antifungal drugs, particularly azole derivatives [6, 7]. Usually, clinical manifestations of fungal infection occur as localized in the mucosa, yet these may become disseminated infections which can involve multiple organs [8]. The reduced number of antifungals available at market, together with the high frequency of their use, has made treatment of fungal infections more complex, since many etiological agents have already acquired resistance to the few drugs available [9–11].

Phenylpropanoic derivatives, especially cinnamic acid derivatives, form a group of substances with varied pharmacological and biological properties. They are found in nature in various forms, such as acids, esters, and amides. Due to their therapeutic potential, studies have been carried out to develop methods of preparation [12]. In addition, certain halogenated derivatives present antifungal activity [13]. Therefore, in the present work we aimed to prepare a collection of twelve esters derived from 4-chlorocinnamic acid to evaluate their antimicrobial potential against the microorganism strains:* Candida albicans *ATCC 90028*; Candida glabrata *ATCC 90030*; Candida krusei *ATCC 34125;* Candida guilliermondii *207;* Pseudomonas aeruginosa *ATCC 8027;* Pseudomonas aeruginosa *102;* Staphylococcus aureus *ATCC 25925; and* Staphylococcus aureus *47. A molecular docking study using the enzyme 14*α*-demethylase as possible site of antifungal action and the structure-activity relationships of the prepared compounds were also performed.

## 2. Experimental Section

### 2.1. Chemistry

#### 2.1.1. Materials

Structural identification of the compounds was performed from ^1^H and ^13^C Nuclear Magnetic Resonance spectra analyses obtained using a MERCURY-VARIAN spectrometer operating at 200 MHz for NMR of ^1^H and 50 MHz for ^13^C NMR, a VARIAN-NMR SYSTEM operating at 500 MHz for NMR of ^1^H and 125 MHz for ^13^C NMR, and an ASCEND-BRUKER system operating at 400 MHz (^1^HNMR) and 100 MHz (^13^CNMR). As the solvent, small amounts of the compounds in deuterated chloroform (CDCl_3_) from MERCK were used. The chemical shifts (*δ*) were expressed in parts per million (ppm) over tetramethylsilane (TMS), which was used as the internal standard. Melting points were recorded on a GEHAKA apparatus, model PF 1500. Infrared spectra were performed using an IRPrestige-21 FTIR spectrophotometer, Shimadzu. Potassium bromide tablets (KBr) were used with frequencies measured in cm^−1^. The unpublished compounds were also analyzed using High Resolution Mass Spectrometry where measurements were performed using a TOF/TOF Ultraflex II mass spectrometer equipped with a high performance solid state laser (*λ* = 355 nm) and reflector. The system was operated using the FlexControl 2.4 (Bruker Daltonics GmbsH, Bremen, Germany) software package. Reactions were monitored and purity was checked using analytical thin-layer chromatography plates.

#### 2.1.2. General Synthesis Method for Ester Derivatives of 4-Chlorocinnamic Acid** 1**-**6**

To a solution of 4-chlorocinnamic acid (0.1 g, 0.547 mmol) in 20 mL of alcohol, 0.2 mL of concentrated sulfuric acid (H_2_SO_4_) was slowly added. The reaction mixture was refluxed with magnetic stirring for 3-24 hours and monitored using silica gel thin-layer chromatography (TLC) and a mixture of hexane and ethyl acetate as eluent. The solvent was partially evaporated by about half, under reduced pressure. Extraction was performed by adding 15 mL of distilled water; the extractive solvent used was ethyl acetate (3 x 10 mL). The resulting organic phases were joined and neutralized with 5% sodium bicarbonate (NaHCO_3_), washed with 10 mL of distilled water, and dried with anhydrous sodium sulfate (Na_2_SO_4_) and then filtered, and the solvent evaporated with a rotary evaporator. For ester** 6**, the purification was carried out using a chromatographic column on silica gel 60 using hexane and ethyl acetate (9:1) as eluents. This procedure was also monitored using TLC [15].

#### 2.1.3. General Method for Synthesis of Esters** 7**–**10**

4-Chlorocinnamic acid (0.1 g, 0.547 mmol) was dissolved in 14 mL of anhydrous acetone. To this solution was added 0.3 mL of triethylamine (2.188 mmol) and halide (0.563 mmol). The flask was then coupled to a reflux condenser. The reaction mixture was refluxed with magnetic stirring for 24-48 hours until consumption of the starting material; this was monitored using TLC. After formation of the product, the solvent was partially evaporated in a rotary evaporator. Subsequently, the reaction product was extracted from 15 mL of distilled water with dichloromethane (3 x 10 mL). The organic phases were joined and treated with 10 mL of 5% sodium bicarbonate (NaHCO_3_). It was then washed with 10 mL of distilled water and dried with anhydrous sodium sulfate (Na_2_SO_4_). Subsequently, filtration was performed and the solvent was evaporated with the aid of a rotary evaporator. The residue was purified using a chromatographic column on silica gel 60 with hexane/ethyl acetate as eluent, in an increasing polar gradient (95:05 – 90:10) [16, 17].

#### 2.1.4. Method for Synthesis of Ester** 11**

4-Chlorocinnamic acid (0.1 g, 0.547 mmol) and perillyl alcohol (0.09 mL, 0.547 mmol) were solubilized in 2 mL tetrahydrofuran (THF). The reaction mixture was placed under magnetic stirring at 0°C for about 30 minutes. Diisopropyl azodicarboxylate (0.12 mL, 0.55 mmol) and triphenylphosphine (0.144 g, 0.547 mmol) were then added, maintaining stirring at room temperature for 72 hours and monitoring with TLC. The solvent was then partially evaporated in a rotary evaporator. Extraction was performed with 10 mL of distilled water and ethyl acetate (3 x 10 mL). The resulting organic layers were joined and neutralized with 5% sodium bicarbonate solution (3 x 10 mL). The reaction mixture was then dried with anhydrous sodium sulfate and filtered, and finally the solvent was evaporated. The product was isolated in a silica gel 60 chromatographic column using hexane/ethyl acetate (9:1) as eluent [18].

#### 2.1.5. Method for Synthesis of Ester** 12**

4-Chlorocinnamic acid (0.1 g, 0.547 mmol), 4-(dimethylamino)pyridine (DMAP) (0.00669 g, 0.0547 mmol), and lauryl alcohol (0.245 mL, 1.095 mmol) were dissolved in dichloromethane (4 mL). Dicyclohexylcarbodiimide (DCC) (0.124 g, 0.602 mmol) dissolved in dichloromethane (6 mL) was then added dropwise. The reaction occurred under magnetic stirring at room temperature and monitored with TLC for 72 hours. After filtration, the reaction product was extracted with 10 mL of distilled water and dichloromethane (3 x 10 mL). The resulting organic phase was treated with 5% hydrochloric acid solution (10 mL). Subsequently a 5% sodium bicarbonate solution (10 mL) was added, followed by 10 mL of distilled water. The solution was dried over anhydrous sodium sulfate, filtered, and rotated to reduce the solvent volume. The product was then purified using column chromatography on silica gel 60 using hexane/ethyl acetate as eluents in increasing order of polarity (100:00-95:05) [19].

*Methyl 4-Chlorocinnamate (* ***1***). White crystals; Yield 97.6% (105.7 mg); m.p.: 71–72°C; IR (KBr, cm^−1^): 3035 (C-H sp^2^), 2949 (C-H sp^3^), 1705 (C=O), 1631 and 1431 (aromatic C=C bending), 1273 and 1166 (C-O stretching), 1004; ^1^H NMR (200 MHz, CDCl_3_): *δ*_H_ 7.61 (*d*,* J* = 16.0 Hz, 1H, H-7); 7.43 (d,* J* = 7.8 Hz, 2H, H-2, H-6); 7.33 (*d*,* J* = 7.8 Hz, 2H, H-3, H-5); 6.38 (*d*,* J* = 16.0 Hz, 1H, H-8); 3.79 (*s*, 3H, H-1′) ppm; ^13^C NMR (50 MHz, CDCl_3_): *δ*_C_ 167.1 (C=O); 143.4 (C-7); 136.2 (C-4); 132.8 (C-1); 129.3 (C-2, C-6); 129.1 (C-3, C-5); 118.2 (C-8); 51.8 (C-1′) ppm.

*Ethyl 4-Chlorocinnamate (* ***2*** *). *Yellow amorphous solid; Yield 89.0% (102.7 mg); IR (KBr, cm^−1^): 3066 (C-H sp^2^), 2981(C-H sp^3^), 1710 (C=O), 1639 and 1448 (aromatic C=C bending), 1311 and 1172 (C-O stretching), 1037; ^1^H NMR (400 MHz, CDCl_3_): *δ*_H_ 7.61 (*d*,* J* = 15.7 Hz, 1H, H-7); 7.43 (*d*,* J* = 8.8 Hz, 2H, H-2, H-6); 7.34 (*d*,* J* = 8.4 Hz, 2H, H-3, H-5); 6.39 (*d*,* J* = 16.0 Hz, 1H, H-8); 4.25 (*q*,* J* = 7.1 Hz, 2H, H-1′); 1.32 (*t*,* J* = 6.9 Hz, 3H, H-2′) ppm; ^13^C NMR (100 MHz, CDCl_3_): *δ*_C_ 166.8 (C=O); 143.3 (C-7); 136.2 (C-4); 133.2 (C-1); 129.3 (C-2, C-6); 129.2 (C-3, C-5); 119.0 (C-8); 60.7(C-1′); 14.4 (C-2′) ppm.

*Propyl 4-Chlorocinnamate (* ***3*** *). *Yellow amorphous solid; Yield 88.5% (109 mg) ); m.p.: 34–36°C; IR (KBr, cm^−1^): 3035 (C-H sp^2^), 2956 (C-H sp^3^), 1705 (C=O), 1635 and 1409 (aromatic C=C bending), 1317 and 1087 (C-O stretching), 1004; ^1^H NMR (200 MHz, CDCl_3_): *δ*_H_ 7.61 (*d*,* J* = 16.0 Hz, 1H, H-7); 7.44 (*d*,* J* = 8.5 Hz, 2H, H-2, H-6); 7.33 (*d*,* J* = 8.6 Hz, 2H, H-3, H-5); 6.40 (*d*,* J* = 16.0 Hz, 1H, H-8); 4.15 (*t*,* J* = 6.7 Hz, 2H, H-1′), 1.71 (*m*,* J* = 7.2 Hz, 2H, H-2′); 0.97 (*t*,* J* = 7.3 Hz, 3H, H-3′) ppm; ^13^C NMR (50 MHz, CDCl_3_): *δ*_C_166.8 (C=O); 143.1 (C-7); 136.1 (C-4); 132.8 (C-1); 129.2 (C-2, C-6); 129.1 (C-3, C-5); 118.7 (C-8); 66.2 (C-1′); 22.0 (C-2′); 10.3 (C-3′) ppm.

*Isopropyl 4-Chlorocinnamate (* ***4*** *). *Yellow solid; Yield 91.2% (112.4 mg); m.p.: 34 – 35°C; IR (KBr, cm^−1^): 3049 (C-H sp^2^), 2980 (C-H sp^3^), 1710 (C=O), 1639 and 1462 (aromatic C=C bending), 1309 and 1109 (C-O stretching); ^1^H NMR (200 MHz, CDCl_3_): *δ*_H_ 7.54 (*d*,* J* = 16.0 Hz, 1H, H-7); 7.36 (*d*,* J* = 7.8 Hz, 2H, H-2, H-6); 7.30 (*d*,* J* = 7.8 Hz, 2H, H-3, H-5), 6.32 (*d*,* J* = 16.0 Hz, 1H, H-8); 5.07 (*m*, 1H, H-1′), 1.25 (*d*,* J* = 5.8 Hz, 6H, H-2′, H-3′) ppm; ^13^C NMR (50 MHz, CDCl_3_): *δ*_C_ 166.2 (C=O); 142.8 (C-7); 135.9 (C-4); 131.0 (C-1); 129.2 (C-2, C-6); 129.1 (C-3, C-5); 119.3 (C-8); 67.9 (C-1′); 22.1 (C-2′, C-3′) ppm.

*Butyl 4-Chlorocinnamate (* ***5*** *). *Brown amorphous solid; Yield 87.4% (115.3 mg); m.p.: 30 - 32°C; IR (KBr, cm^−1^): 3034 (C-H sp^2^), 2960 (C-H sp^3^), 1712 (C=O), 1637 and 1473 (aromatic C=C bending), 1313 and 1172 (C-O stretching), 1010; ^1^H NMR (200 MHz, CDCl_3_): *δ*_H_ 7.61 (*d*,* J* = 16.0 Hz, 1H, H-7); 7.44 (*d*,* J* = 7.9 Hz, 2H, H-2, H-6); 7.34 (*d*,* J* = 7.9 Hz, 2H, H-3, H-5); 6.40 (*d*,* J* = 16.0 Hz, 1H, H-8); 4.20 (*t*,* J* = 6.2 Hz, 2H, H-1′); 1.69 (*quint*,* J* = 6,7 Hz, 2H, H-2′); 1.42 (*sex*,* J* = 7.1 Hz, 2H, H-3′); 0.95 (*t*,* J* = 7,0 Hz, 3H, H-4′) ppm; ^13^C NMR (50 MHz, CDCl_3_):  *δ*_C_166.8 (C=O); 143.1 (C-7); 136.0 (C-4); 132.9 (C-1); 129.2 (C-2, C-6); 129.1 (C-3, C-5); 118.8 (C-8); 64.5 (C-1′); 30.7 (C-2′); 19.2 (C-3′); 13.7 (C-4′) ppm.

*2-Methoxyethyl 4-Chlorocinnamate (* ***6*** *). *Yellow oil; Yield 36.0% (47.6 mg); IR (KBr, cm^−1^):3068 (C-H sp^2^), 2927 (C-H sp^3^), 1714 (C=O), 1639 and 1452 (aromatic C=C bending), 1313 and 1170 (C-O stretching), 1039; ^1^H NMR (400 MHz, CDCl_3_): *δ*_H_ 7.67 (*d*,* J* = 16.0 Hz, 1H, H-7); 7.46 (*d*,* J* = 8.8 Hz, 2H, H-2, H-6); 7.36 (*d*,* J* = 9.7 Hz, 2H, H-3, H-5), 6.47 (*d*,* J* = 16.0 Hz, 1H, H-8); 4.38 (*t*,* J* = 5.4, 2H, H-1′); 3.68 (*t*,*J* = 4.7, 2H, H-2′); 3.43 (*s*, 3H, H-3′) ppm; ^13^C NMR (100 MHz, CDCl_3_): *δ*_C_166.8 (C=O); 143.8 (C-7); 136.3 (C-4); 133.0 (C-1); 129.4 (C-2, C-6); 129.3 (C-3, C-5); 118.5 (C-8); 70.64 (C-1′); 63.7 (C-2′); 59.1 (C-3′) ppm; HRMS (MALDI) calculated or C_12_H_13_ClO_3_[M + Na]^+^: 263.6722; encountered: 263.6333.

*Pentyl 4-Chlorocinnamate (* ***7*** *). *White amorphous solid; Yield 55.6% (77 mg); m.p.: 38 – 39°C; IR (KBr, cm^−1^): 3035 (C-H sp^2^), 2958 (C-H sp^3^), 1703 (C=O), 1635 and 1492 (aromatic C=C bending), 1311 and 1172 (C-O stretching), 1083; ^1^H NMR (400 MHz, CDCl_3_): *δ*_H_ 7.61 (*d*,* J* = 16.0 Hz, 1H, H-7); 7.44 (*d*,* J* = 8.6 Hz, 2H, H-2, H-6); 7.34 (*d*,* J* = 8.5 Hz, 2H, H-3, H-5 ); 6.40 (*d*,* J* = 16.0 Hz, 1H, H-8); 4.19 (*t*,* J* = 6.8 Hz, 2H, H-1′); 1.69 (*quint*,* J* = 6.9 Hz, 2H, H, 2′); 1.37 (*m*, 4H, H-3′, H-4′), 0.92 (*t*,*J*= 7.0 Hz, 3H, H-5′) ppm; ^13^C NMR (100 MHz, CDCl_3_): *δ*_C_166.8 (C=O); 143.1 (C-7); 136.1 (C-4); 133.0 (C-1); 129.2 (C-2, C-6); 129.1 (C-3, C-5); 118.9 (C-8); 63.8 (C-1′); 28.4 (C-2′); 28.1 (C-3′); 22.4 (C-4′); 14.0 (C-5′) ppm.

*Decila 4-Chlorocinnamate (* ***8*** *). *Yellow oil; Yield 30.6% (54.2 mg); IR (KBr, cm^−1^):3066 (C-H sp^2^), 2926 (C-H sp^3^), 1710 (C=O), 1637 and 1465 (aromatic C=C bending), 1311 and 1168 (C-O stretching), 1012; ^1^H NMR (400 MHz, CDCl_3_): *δ*_H_ 7.62 (*d*,* J* = 16.0 Hz, 1H, H-7); 7.45 (*d*,* J* = 8.4 Hz, 2H, H-2, H-6); 7.35 (*d*,* J* = 8.5 Hz, 2H, H-3, H-5); 6.41 (*d*,* J* = 16.0 Hz, 1H, H-8); 4.19 (*t*,* J* = 6,7 Hz, 2H, H-1′); 1.68 (*quint*,* J* = 6.6 Hz, 2H, H-2′), 1.40 – 1.24 (*m*, 14H, H-3′, H-4′, H-5′, H-6′, H-7′, H-8′, H-9′); 0.88 (*t*,* J* = 6.7 Hz, 3H, H-10′) ppm.^13^C NMR (100 MHz, CDCl_3_): *δ*_C_166.9 (C=O); 143.1 (C-7); 136.0 (C-4); 133.0 (C-1); 129.2 (C-2, C-6); 129.1 (C-3, C-5); 118.7 (C-8); 64.8 (C-1′); 32.0 (C-2′); 29.5 (C-3′); 29.3 (C-4′); 29.2 (C-5′); 28.7 (C-6′); 26.0 (C-7′); 22.7 (C-8′, C-9′)*∗*; 14.0 (C-10′) ppm.

*∗*Chemical shift for carbons.

*4*′*-Chlorobenzyl 4-Chlorocinnamate ( ****9***). White solid; Yield 61.6% (103.5 mg);m.p.: 131 – 133°C; IR (KBr, cm^−1^): 3057 (C-H sp^2^), 2949 (C-H sp^3^), 1701 (C=O), 1653 and 1450 (aromatic C=C bending), 1273 and 1188 (C-O stretching), 1010 (C-Cl stretching); ^1^H NMR (500 MHz, CDCl_3_): *δ*_H_ 7.66 (d,* J* = 16.0 Hz, 1H, H-7); 7.44 (d,* J* = 8.8 Hz, 2H, H-2, H-6), 7.36 (d,* J* =7.1 Hz, 6H, H-3, H-5, H-3′, H-4′, H-6′, H-7′); 6.44 (d,* J* = 16.0 Hz, 1H, H-8); 5.21 (s, 2H, H-1′) ppm;^13^C NMR (125 MHz, CDCl_3_): *δ*_C_166.3 (C=O); 144.0 (C-7); 136.3 (C-2′); 134.5 (C-4); 134.2 (C-1); 132.7 (C-5′); 129.6 (C-3′, C-7′); 129.2 (C-2, C-6); 129.2 (C-4′, C-6′); 128.8 (C-3, C-5); 118.2 (C-8); 65.6 (C-1′) ppm; HRMS (MALDI) calculated for C_16_H_12_Cl_2_O_2_[M +Na]^+^: 330.161; encountered 330.1609.

*4*′*-Methoxybenzyl 4-Chlorocinnamate ( ****10***). White amorphous solid*; *Yield 39.3% (65.1 mg); m.p.: 80 – 83°C; IR (KBr, cm^−1^): 3035 (C-H sp^2^), 2960 (C-H sp^3^), 1712 (C=O), 1610 and 1442 (aromatic C=C bending), 1261 and 1159 (C-O stretching), 1014; ^1^H NMR (500 MHz, CDCl_3_): *δ*_H_ 7.66 (*d*,* J* = 16.5 Hz, 1H, H-7); 7.44 (*d*,* J* = 8.5 Hz, 2H, H-2, H-6); 7.35 (*d*,* J* = 5.1 Hz, 4H, H-3, H-5, H-2′, H-6′); 6.92 (*d*,* J* = 8.7 Hz, 2H, H-3′, H-5′); 6.44 (*d*,* J* = 16.0 Hz, 1H, H-8); 5.19 (*s*, 2H, H-7′), 3.82 (*s*, 3H, H-8′) ppm; ^13^C NMR (125 MHz, CDCl_3_): *δ*_C_166,6 (C=O); 159.7 (C-4′); 143.5 (C-7); 136.2 (C-4); 132.9 (C-1); 130.1 (C-2′, C-6′);129.2 (C-2, C-6); 129.1 (C-3, C-5); 128.1 (C-1′); 118.6 (C-8); 114.0 (C-3′, C-5′); 66.3 (C-7′); 55.1 (C-8′) ppm.

*Perillyl 4-Chlorocinnamate (* ***11***). Yellow oil; Yield 30.7% (53.6 mg); IR (KBr, cm^−1^): 3070 (C-H sp^2^), 2926 (C-H sp^3^),1712 (C=O), 1637 and 1406 (aromatic C=C bending), 1273 and 1166 (C-O stretching), 1012; ^1^H NMR (400 MHz, CDCl_3_): *δ*_H_ 7.64 (*d*,* J* = 16.0 Hz, 1H, H-7); 7.45 (*d*,* J* = 8.4 Hz, 2H, H-2, H-6); 7.35 (*d*,* J* = 8.5 Hz, 2H, H-3, H-5); 6.43 (*d*,* J* = 16.0 Hz, 1H, H-8); 5.81 (*s*, 1H, H-2′); 4.73 (*s*, 2H, H-10′), 4.60 (*s*, 2H, H-7′); 2.20 (*m*, 1H, H-3′); 2.16 (*m*, 1H, H-4′); 2.15 – 2.10 (*m*, 2H, H-6′); 2.03 – 1.96 (*m*, 1H, H-3′); 1.90 – 1.84 (*m*, 1H, H-5′); 1.76 – 1.72 (*m*, 3H, H-9′), 1.56 – 1.46 (*m*, 1H, H-5′) ppm; ^13^C NMR (100 MHz, CDCl_3_): *δ*_C_ 166.7 (C=O); 149.6 (C-8′); 143.4 (C-7); 136.2 (C-4); 132.9 (C-1); 132.6 (C-1′); 129.2 (C-2, C-6); 129.1 (C-3, C-5); 126.0 (C-2′); 118.7 (C-8); 108.5 (C-10′); 68.7 (C-7′); 40.8 (C-4′); 30.5 (C-3′); 27.2 (C-5′); 26.4 (C-6′); 20.6 (C-9′) ppm; HRMS (MALDI) calculated for C_19_H_21_ClO_2_ [M + Na]^+^: 339.8121; encountered 339.8119.

*Dodecyl 4-Chlorocinnamate (* ***12***). Colorless oil; Yield 26.3% (115.2 mg); IR (KBr, cm^−1^): 3068 (C-H sp^2^), 2926 (C-H sp^3^), 1714 (C=O), 1639 and 1465 (aromatic C=C bending), 1271 and 1168 (C-O stretching), 1012; ^1^H NMR (400 MHz, CDCl_3_): *δ*_H_ 7.62 (*d*,* J* = 16.0 Hz, 1H, H-7); 7.45 (*d*,* J* = 8.4 Hz, 2H, H-2, H-6); 7.35 (*d*,* J* = 8.5 Hz, 2H, H-3, H-5); 6.41 (*d*,* J* = 16,0 Hz, 1H, H-8); 4.20 (*t*,* J* = 6,7 Hz, 2H, H-1′); 1.69 (*quint*,* J* = 6.7 Hz, 2H, H-2′); 1.26 (*m*, 8H, H-3′, H-4′, H-5′, H-6′, H-7′, H-8′, H-9′, H-10′, H-11′); 0.89 (*t*,* J* = 6.7 Hz, 3H, H-12′) ppm; ^13^C NMR (100 MHz, CDCl_3_): *δ*_C_166.9 (C=O); 143.1 (C-7); 136.1 (C-4); 133.0 (C-1); 129.2 (C-2, C-6); 129.2 (C-3, C-5); 118.9 (C-8); 64.9 (C-1′); 31.9 (C-2′); 29.7 (C-3′); 29.6 (C-4′); 29.6 (C-5′); 29.5 (C-6′); 29.4 (C-7′); 29.3 (C-8′); 28.7 (C-9′); 25.9 (C-10′); 22.7 (C-11′); 14.2 (C-12′) ppm.

### 2.2. Antimicrobial Activity

#### 2.2.1. Antifungal Activity

The antifungal activity of the prepared compounds was assessed against four selected fungus groups: reference strains of* Candida *spp. which were obtained from the American Type Culture Collection (ATCC),* C. albicans* ATCC 90028;* C*.* glabrata* ATCC 90030;* C. krusei* ATCC 34125; and* C. guilliermondii *ATCC 22017.

*(1) Compound Preparation for Testing*. To analyze antifungal activity in fungal yeast strains, the compounds were submitted to biological assays. They were solubilized in dimethylsulfoxide (DMSO) (final concentration ≤ 5%) and then in sterile distilled water (completing to 1.0 mL) [20–23].

*(2) Determination of Minimum Inhibitory Concentrations (MICs)*. The MIC was determined using the microdilution technique, as previously described by the CLSI [24]. Briefly, microtiter plates with 96 U-bottom wells were used, and serial dilutions of the test substance, culture medium, and fungal inoculum (2.5 10^3^ CFU/mL, 530 nm, abs 0.08 to 0.1) were added to the plates. The plates were incubated for 24 h at 35°C, and the results were read by visual observation of cell aggregates at the bottom of the wells. Nystatin (Sigma-Aldrich, São Paulo, SP) was used as a positive control. Controls for strain viability and medium sterility, DMSO (dimethyl sulfoxide) (Sigma-Aldrich, São Paulo, Brazil) used for compound preparation, were performed simultaneously with the assay. TTC (2,3,5-triphenyl tetrazolium chloride) dye was added to each well in order to confirm the presence of viable microorganisms [25]. The MIC was defined as the lowest concentration of the test substance inhibiting microbial growth.

*(3) Determination of the Minimum Fungicide Concentrations (MFCs)*. Onto Petri dishes containing Sabouraud Dextrose Agar (SDA) (KASVI®, KasvImport and Distribution of Laboratory Products LTDA, Curitiba, Brazil) 50 *μ*L well aliquots corresponding to MIC and two concentrations above (2 MIC and 4 MIC) were plated. The plates were incubated for 24 h at 35°C, and the reading was performed by visual observation of fungal growth on the solid medium. The MFC was defined as the lowest concentration inhibiting visible growth on solid medium [26]. The MFC/MIC ratio was calculated to determine whether the substance presented fungistatic (MFC/MIC ≥ 4) or fungicidal (MFC/MIC < 4) activity [14].

#### 2.2.2. Antibacterial Activity

Antibacterial activity was assessed for the prepared compounds against Gram-positive and Gram-negative bacteria from the American Type Culture Collection (ATCC) and also from clinical origins, namely,* Pseudomonas aeruginosa *ATCC 8027;* Pseudomonas aeruginosa *102;* Staphylococcus aureus *ATCC 25925; and* Staphylococcus aureus *47. The bacteria were cultured in a medium consisting of yeast extract (HIMEDIA) 5 g, peptone (MERCK) 10 g, NaCl (MERCK) 5 g, KH_2_ PO_4_ (MERCK) 1.5 g, and (NaHPO_4_) 12H_2_O (MERCK) 9 g solubilized in 1 L of distilled water and sterilized using autoclaving at 121°C and 1 atm for 20 minutes.

*(1) Compound Preparation for Testing. *To prepare the twelve esters for antibacterial testing, a 10 mg/ml solution of each of the substances was made using 25% DMSO in sterile distilled water.

*(2) Determination of Minimum Inhibitory Concentration (MIC*). MIC determination was performed using microdilution technique and 96-well plates for each of the strains tested, as described by Gerhardt et al. (1994) [27]. For this, 100 *μ*L of the culture medium was distributed to all of the wells, except those of the first and second columns that received 150 *μ*L. Subsequently, 50 *μ*L of the 10 mg/mL solution of each of the substances was added to the 1st and 2nd well columns. From the second column serial dilution was performed in halves, obtaining the final concentrations of 500-15.6 *μ*g/mL for each of the substances tested. The volume was completed with 100 *μ*L of the bacterial culture making up the final volume at 200 *μ*L. To test 1000 *μ*g of each of the substances, larger volumes (1 mL) were used in Eppendorf tubes to keep DMSO to a concentration which would not affect the bacteria (≤ 8%).

The plates and tubes were incubated at 37°C for 24 h for further reading, which was performed with the addition of 20 *μ*L of a 0.01% (w/v) solution of resazurin sodium (SIGMA), a colorimetric indicator of metabolic activity. The MIC was considered to be the lowest concentration that completely inhibited bacterial growth.

### 2.3. Docking Study

The experimental* in vitro* anti-*Candida *activity of 4-chlorocinnamic acid derivatives was explored and correlated via molecular docking study, which involves study of possible interactions with cytochrome P450 14*α*-sterol demethylase from* C. albicans *(EC:1.14.13.70;* Candida *P450DM).

#### 2.3.1. Ligand Preparation

Chem sketch (Version 12.01) was used for the generation of 2D structure of the synthesized derivatives which were further converted to 3D structures. The structures were further energetically minimized and saved in MDL MolFiles.

#### 2.3.2. Docking Procedure

Molegro Virtual Docker (MVD 2010.4.1.0) program was used for the docking analysis of the synthesized compounds using cytochrome P450 14*α*-demethylase from* Candida albicans* (*Candida *P450DM) with ID 1EA1. The hydrogen atoms were added in the structure followed by assigning bond orders. Further, the potential binding sites were determined by refining the structure using grid based cavity prediction algorithm. The site possessing highest number of amino acid residues, i.e., 189.44 amino acids, and nearest to heme cofactor was chosen as active site [28].

## 3. Results and Discussion

### 3.1. Chemistry

**Scheme 1:**
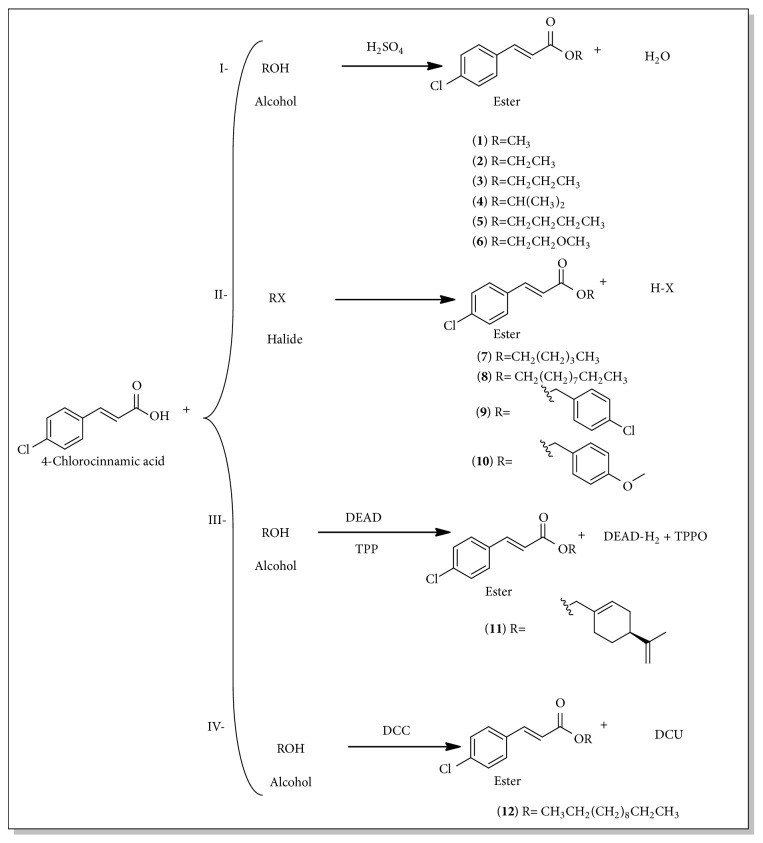
Esterification reactions: (*I*) Fischer's esterification; (*II*) bimolecular nucleophilic substitution (S_N_2) with halides; (*III*) Mitsunobu reaction; (*IV*) Steglich reaction.

Compounds** 1-6** were prepared by Fischer esterification, and reaction times were from 3-24 hours, with satisfactory yields (36.0-97.6%). For compounds** 7-10** the alkyl or aryl halide esterification methods were employed, and reactions times were from 24 to 48 hours with yields of 30.6-61.6%. Compound** 11** was prepared via the Mitsunobu reaction, with a reaction time of 72 hours and a yield of 30.7%. The Steglich esterification methodology was used to obtain the ester** 12**; reaction time was 72 hours with a yield of 26.3% (Scheme 1).

The infrared spectra of the 4-chlorocinnamic acid analogs presented absorption bands at 3000 relative to the C-H sp^2^ stretch, with C=C ring stretch absorption bands occurring in pairs in the 1600 and 1475 cm^−1^ regions; C=O stretch bands occurred in the 1750-1735 cm^−1^ range; C-O stretch assigned to C-O bonds in the ester appears with two bands in the range of 1300 to 1000 cm^−1^; and C-Cl stretch occurred in the 1010-1000 range. Alkyl analogs exhibit alkane (sp^3^) C-H stretching bands at about 3000 cm^−1^, methylene groups with angular deformation absorption at 1465 cm ^−1^, and methyl groups at 1375 cm^−1^. In aryl derivatives, a strong folding vibration band was found at the* para *position in the region of 800-850 cm^−1^, a characteristic absorption for its* para*-substituent.

In ^1^H and ^13^C NMR of the prepared products, six hydrogens can be observed in common (H-2, H-3, H-5, H-6, H-7, and H-8), where four are from the aromatic ring and two from the side chain attached to the carbonyl. In the hydrogen (CDCl_3_, 400 MHz, ppm) spectral data for ester** 6** olefinic hydrogens were observed which are presented as two doublets in *δ* 7.67 (d,* J* = 16.0 Hz, 1H) and 6.47 (d,* J* = 16.0 Hz, 1H), given, respectively, to the hydrogens H-7 and H-8; the coupling constant is equal (*J* = 16.0 Hz). For the aromatic hydrogens we observed two doublets each, referring to two hydrogens 7.46 (d,* J* = 8.8 Hz, 2H) and 7.36 (d,* J* = 9.7 Hz, 2H), belonging to hydrogens H-2, H-6, H-3, and H-5, respectively.

There were also nine carbons in common for all of the analogs (C-1, C-2, C-3, C-4, C-5, C-6, C-7, C-8, C=O), six from the aromatic ring and three from the side chain with the presence of the ester carbonyl. In the ^13^C NMR (CDCl_3_, 400 MHz, ppm) spectral data of ester** 6** presented peaks at 166.8 (C=O of esters), 143.8 from C-7, 136.3 from C-4, and 133.0 attributed to carbon C-1 and a peak at 129.4 corresponding to carbons C-2 and C-6 and also occurring for the 129.3 peaks of carbons C-3 and C-5. The peak at 118.5 refers to the C-8.

### 3.2. Biology

#### 3.2.1. Bioactivity of the Tested Compounds against Pathogenic Fungi of the Candida Genus

The microorganisms used in this study are associated with infections in humans and are related to high rates of morbidity and mortality, especially in debilitated and immunocompromised individuals.* C. albicans* is the most common species in patients with fungal infections and presents a remarkable ability to develop virulence factors while* C. glabrata* and* C. krusei* are especially resistant to azole antifungals [29, 30].* C. guilliermondii* has been reported in patients diagnosed with candidemia [31].

Twelve ester derivatives of 4-chlorocinnamic acid were prepared and tested against strains of the genus* Candida*. Analysis of the structure-activity relationships of 4-chlorocinnamic acid-derived esters was based on the minimum inhibitory concentration (MIC) results against the* Candida* genus fungal strains tested. The preparation of the structurally similar compounds and changes in the size of the alkyl group or the types of substituents on the aromatic ring (such as electron donors or attractors) can promote significant changes in the properties of the compounds and thus result in distinct antifungal potencies.

The antifungal activity results for esters** 1** to** 12** under the CLEELAND, SQUIRES (1991), and NCCLS/CLSI (2002) [20, 24] protocols are presented in Tables 1 and 2. All of the compounds presented bioactivity; however some did not present bioactivity against all of the strains tested. Regarding the assays against strains of* Candida albicans* ATCC 90028 and according to the results presented in Table 1, esters** 1**,** 2**,** 6**,** 8**,** 11,** and** 12** were bioactive; the others were inactive. It was observed that the ester methyl 4-chlorocinnamate (**1**), structurally the simplest ester of all the preparations (MIC = 5.09 *μ*mol/mL), presented antifungal activity at its highest tested concentration, an activity reported by [32]. Substitution using an ethyl group produced a slight increase in antifungal activity against this strain. Compound ethyl 4-chlorocinnamate (**2**) presented a MIC = 4.75 *μ*mol/mL, as compared with compound** 1**, presenting slightly higher activity. There was loss of biological activity when increasing alkyl side chain length as seen in esters** 3**,** 4,** and** 5**, which suggests that increasing alkyl side chain length when formed by three or four carbon atoms results in inactivity.

The ester 2-methoxyethyl 4-chlorocinnamate (**6**) contains in its side chain a heteroatom presenting a MIC = 2.08 *μ*mol/mL. When comparing this compound with ester** 2** it can be inferred that the presence of a heteroatom in the lateral alkyl chain results in better bioactivity. No activity was observed for the ester pentyl 4-chlorocinnamate (**7**), in which the lateral alkyl chain was lengthened, formed by five carbon atoms. An increase in lipophilicity resulted in the inactivity for the molecule. Yet with a ten-carbon chain, the ester decyl 4-chlorocinnamate (**8**) at its highest concentration, MIC = 3.10 *μ*mol/mL, inhibited growth of the tested strain. Comparing compound** 8** with 4′-chlorobenzyl 4-chlorocinnamate (**9**) and with 4′-methoxybenzyl 4-chlorocinnamate (**10**) which presented no bioactivity suggests that exchanging a ten-carbon chain with an aromatic ring does not result in antifungal activity.

The perillyl 4-chlorocinnamate ester (**11**) presented a MIC = 1.58 *μ*mol/mL, the best result against the* C. albicans* tested strain. This is due to the presence of the terpenic substructure. It is reported that terpenes present a good antifungal profile [33–35]. Comparing ester** 11 **to dodecyl 4-chlorocinnamate (**12**), MIC = 2.85 *μ*mol/mL, it can be inferred that exchanging an unsaturated alicyclic with a saturated acyclic chain diminishes the bioactivity of the compound. According to the results of the MFC/MIC ratio esters** 1**,** 2**,** 6**,** 8**,** 11**, and** 12 **possess fungicidal activity, as seen in Table 2.

Considering the results against* Candida glabrata* strain (ATCC 90030) of the twelve esters tested, compounds** 1**,** 2**,** 5**,** 6**,** 7**,** 8**,** 11,** and** 12 **present bioactivity against the strain. Using compound** 1** as a base, presenting fungal inhibition at its highest concentration tested (MIC = 5.09 *μ*mol/mL), compound** 2** was also bioactive at its highest concentration tested (MIC = 4.75 *μ*mol/mL). Yet side chains containing three carbon atoms such as in esters** 3** and** 4**, respectively, either straight or branched chains, present no bioactivity.

Comparing the nonactive propyl 4-chlorocinnamate (**3**) with butyl 4-chlorocinnamate (**5**) it can be inferred that the slight increase of CH_2_ in the side chain of compound** 5 **provided bioactivity, suggesting that the increase from three to four carbon atoms in the side chain resulted in increased lipophilicity; i.e., the substance may bind to cell membrane sterols and thus cause outflows of cellular constituents and even cell death. This assertion, however, applies only to specific carbon chain lengths, depending on several factors involving both the molecule and the microorganism tested.

Considering ester** 5 **as compared to ester** 6**, it may be suggested that the presence of a heteroatom such as an oxygen on the side chain of the compound results in greater bioactivity, since compound** 6** presented a MIC = 2.08 *μ*mol/mL, being 1/2 that of** 5** (MIC = 4.19 *μ*mol/mL), thus more potency. The activity decreases when a heteroatom containing side chain is exchanged for chains with five or ten carbons, as was the case for compounds** 7** and** 8** (presenting MICs = 3.96 *μ*mol/mL and 3.10 *μ*mol/mL, respectively), suggesting that the presence of an oxygen in the side chain of the ester provides it with a better antifungal profile.

Comparing compound** 8** with the ester 4-chlorobenzyl 4-chlorocinnamate (**9**) and with 4-methoxybenzyl 4-chlorocinnamate (**10**), the two aromatic esters were inactive against this* C. glabrata *strain. When comparing to alkyl esters, we infer that the aryl esters tested for presenting a voluminous group close to the carbonyl present no antifungal activity; interaction of the aryl esters with their biological target is more difficult. When comparing compound** 11** (presenting the best bioactivity in relation to the* C. glabrata* strain and obtaining a MIC = 1.58 *μ*mol/mL) to compound** 12 **(presenting decreased activity) and exchanging an unsaturated alicyclic substituent (a terpenic substructure) with a saturated acyclic chain, compound** 12 **bioactivity decreased, presenting a MIC of 2.85 *μ*mol/mL. For this* C. glabrata* strain, the compounds that presented bioactivity were** 1**,** 2**,** 5**,** 6**,** 7**,** 8**,** 11,** and** 12**, all of which presented the ability to inhibit the growth of the strain as well as exhibiting fungicidal activity according to their MFC/MIC ratio.

Of the results involving the* Candida krusei* strain (ATCC 34135), the compounds that presented bioactivity were** 2**,** 6**,** 8**,** 11**, and** 12**. Comparing compounds** 1 **and** 2**, compound** 1 **presented no activity against this strain. Addition of a methylene group in the side chain conferred bioactivity (at highest concentration for compound** 2**). However, carbon chain increases such as in** 3**,** 4**, and** 5** resulted in inactivity against this* C. krusei *strain. When a heteroatom was added to the molecule as in compound** 6**, bioactivity was observed at the highest concentration tested, MIC = 4.16 *μ*mol/mL. Such activity does not occur when a lateral chain formed by five carbon atoms is present, as in compound** 7**, which is inactive against the* C. krusei* strain. Comparing** 7** and** 8** it can be observed that substitution of a five-carbon atom side chain with a ten-carbon atom side chain yields antifungal activity MIC = 3.10 *μ*mol/mL. While comparing ester** 8** with** 9** and** 10**, there was loss of bioactivity (as occurred in the two previous strains). Exchanging a linear alkyl side chain using aryl groups resulted in an inactive compound. Analyzing compounds** 10** and** 11** against the* C. krusei *strain, when exchanging an aryl substituent with an unsaturated alicyclic substituent (for compound** 11**; MIC = 0.78 *μ*mol/mL), the derivative presented the best antifungal activity of all the esters tested. In analyzing esters** 11** and** 12**, it was observed that substitution of the unsaturated alicyclic chain with a linear saturated acyclic chain generates diminished biological potency (about three times smaller). The presence of a terpenic substructure potentiates antifungal action. In relation to the* C. krusei* strain tested, the esters that presented activity were** 2**,** 6**,** 8**,** 11**, and** 12**. In addition, inhibiting fungal growth, they obey the MFC/MIC ratio to present fungicidal activity.

In the present work,* Candida guilliermondii *ATCC 22017 was the most susceptible microorganism; thus all of the esters tested were bioactive. Esters** 1** (MIC = 1.27 *μ*mol/mL) and** 2** (MIC = 1.19 *μ*mol/mL) presented similar antifungal potency. Comparing compound** 2 **with** 3**,** 4**, and** 5**, we observed a decline in activity, MIC = 2.22 *μ*mol/mL, for compounds** 3** and** 4**, and for compound** 5** the MIC = 2.09 *μ*mol/mL. Comparing compound** 5** with** 6**, we note a bioactivity increase (lowering MIC to 0.13 *μ*mol/mL), which is the second best result of the study. It can be inferred that the exchange of a methylene group with oxygen gives the molecule greater antifungal potency, possibly by increasing electronic affinity, which can be considered a main factor in determining better antimicrobial activity [36].

Analyzing ester compound** 6 **with compounds** 7** and** 8**, it was observed that more extensive carbon side chains (of five and ten carbon atoms, respectively) and absence of a heteroatom diminished compound activity, MICs of 1.98 *μ*mol/mL and 1.55 *μ*mol/mL, respectively. Comparing compound** 8 **with** 9** and** 10**, it was observed that converting an extended alkyl chain to* para-*chlorinated aromatic ring or to* para*-methoxylated aromatic ring yields molecules with better bioactivity (MICs = 0.40 and 0.41 *μ*mol/mL, respectively), roughly four times more active than compound** 8**.

Ester** 11** presented the best activity of all the tested compounds, obtaining a MIC = 0.024 *μ*mol/mL against the* C. guilliermondii *strain. This may be due to the fact that the substance presents a terpenic substructure, which conferred greater antifungal activity than all of the other tested compounds. It has been reported that terpenes present good antifungal profiles [33–35]. Comparing the results of** 11 **and** 12**, the latter presenting a MIC = 1.42 *μ*mol/mL, it was noted that the presence of a terpenic group made the compound more active against the* C. guilliermondii *strain, than if a saturated acyclic side chain was present. Thus, analyzing the results against the* C. guilliermondii *strain, it was observed that all twelve esters presented inhibitory activity. However, compounds** 7** and** 12** presented no fungicidal activity (Table 2).

#### 3.2.2. Bioactivity of Tested Compounds against the Pathogenic Bacteria.

The use of* Pseudomonas aeruginosa* and* Staphylococcus aureus* in this study is justified because they are associated with infection in a hospital environment [37]. According to the antibacterial assays performed with the twelve esters derived from 4-chlorocinnamic acid, only the ester methyl 4-chlorocinnamate (**1**) was able to inhibit the growth of the* S. aureus *ATCC 25925 strain (at the highest concentration tested), MIC = 5.09 *μ*mol/mL. It has been reported by TONARI et al. (2002) [38] that 4-chlorocinnamic acid presents activity against bacterial strains. However, a slight increase in the lipophilicity of the molecule resulted in activity loss; for esters** 2 **through** 12**, inhibitory action was not observed, as seen in Table 3.

### 3.3. Molecular Modelling

The antifungal potency of the 4-chlorocinnamic acid esters was investigated using the chimeric enzyme. The modeled structure was energetically minimized and was subjected to molecular docking analysis using Molegro Virtual Docker (MVD 2010.4.1.0).

The chimeric enzyme along with ligand, heme cofactor, and water molecules was imported in the MVD software. The site having ligand, nearer to heme cofactor, was preferred as the best region for the binding of the prepared compounds. Docking was carried out involving 10 independent docking runs for each compound on the active site of 14*α*-demethylase [28].

**Figure 1:**
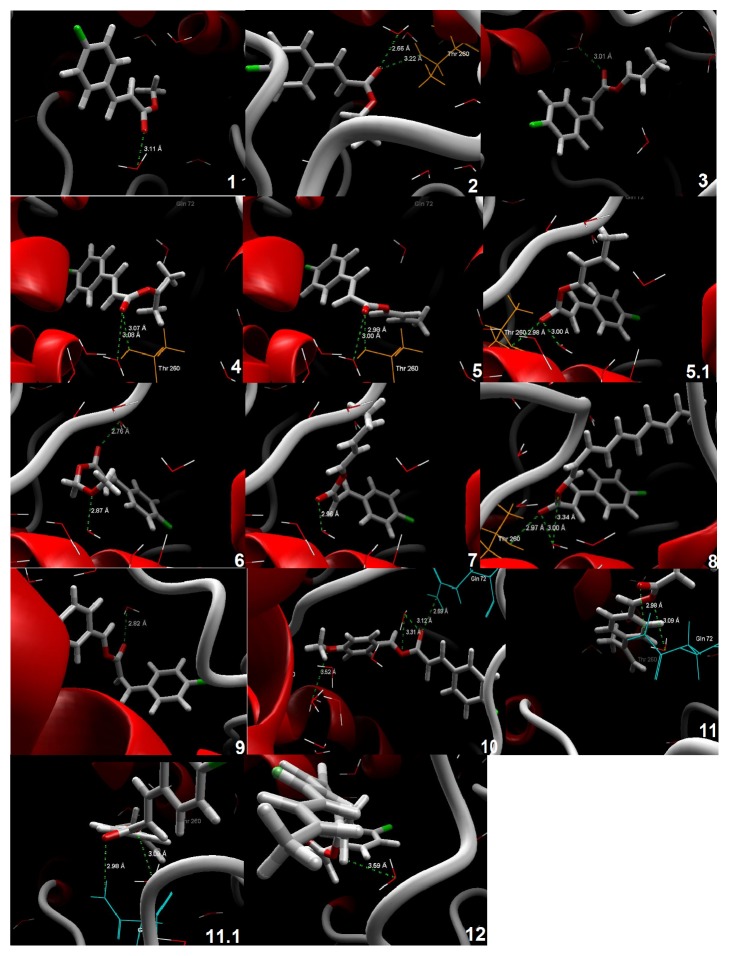
Binding mode of compounds** 1**-**12 **in the active site of cytochrome P450 14*α*-demethylase of* C. albicans*.

The protein-ligand interaction was screened via the docking score function, i.e., Mol Dock Score, PLANTS Score, and Rerank Score. The predicted binding energy and other docking results of the esters are tabulated in Table 4 and Figure 1.

**Figure 2:**
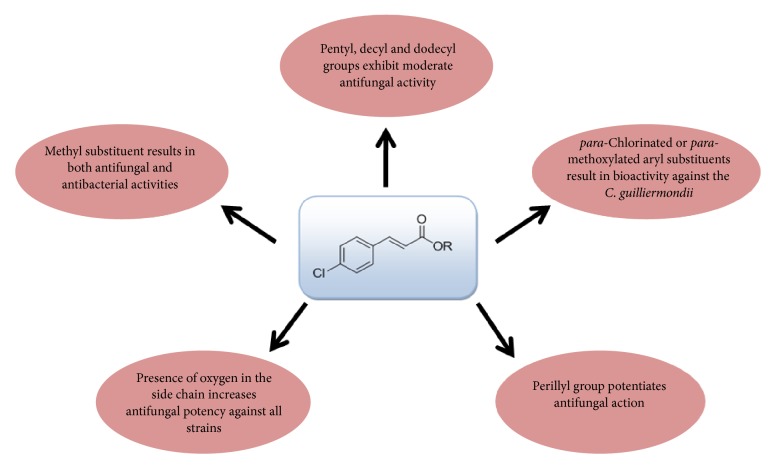
Main relationships between chemical structure and antimicrobial activity of esters** 1-12**.

The docking scores indicated that the esters can act as potential inhibitors of 14*α*-demethylase enzyme. The molecular modeling studies depicted that most of the synthesized derivatives exhibited interaction with the protein residue and two protein residues were necessary for ligand binding, namely, Gln 72 and Thr 260. The protein-ligand interaction is attributed to the binding potency of ligand with the protein. The molecular docking results confirmed the interactions of synthesised 4-chlorocinnamic acid esters with the active site of cytochrome P450 14*α*-demethylase and depicted that the esters can be further explored for antifungal potency. In fact, the use of this enzyme as a possible site of antifungal action has virtually confirmed the bioactivity of several chemical classes, for example, 2-mercaptoimidazoles [28].  Figure 2 shows a summary of the main aspects of the antimicrobial structure-activity relationship of the twelve esters tested.

## 4. Conclusion

Of the twelve esters prepared, only the structurally simpler compound, methyl 4-chlorocinnamate (**1**), presented activity against* S. aureus* strain ATCC 25925 at the highest concentration tested. For antifungal activity, all of the compounds were active. Considering the four strains tested:* Candida albicans *ATCC 90028*, Candida glabrata *ATCC 90030,* Candida krusei *ATCC 34125 and* Candida guilliermondii* 207, compounds** 6** and** 11 **presented good activity against all strains tested as highlighted, especially for* C. guilliermondii* 207. The molecular modeling study revealed good affinity of the esters with the possible biological target related to anti-*Candida* activity. The presence of a heteroatom in the carbon chain, or a terpenic substructure, results in esters with better antifungal profiles. Thus, esters prepared from 4-chlorocinnamic acid may be employed for antifungal compound studies.

## Figures and Tables

**Table 1:** Minimum inhibitory concentration MIC (*μ*mol/mL) of compounds **1-12** against yeasts of the *Candida *genus, Microdilution Technique.

Compounds	*Candida albicans*	*Candida glabrata*	*Candida krusei*	*Candida guilliermondii*
	ATCC 90028	ATCC 90030	ATCC 34135	ATCC 22017
**1**	5.09 *μ*mol/mL	5.09 *μ*mol/mL	+	1.27 *μ*mol/mL

**2**	4.75 *μ*mol/mL	4.75 *μ*mol/mL	4.75 *μ*mol/mL	1.19 *μ*mol/mL

**3**	+	+	+	2.22 *μ*mol/mL

**4**	+	+	+	2.22 *μ*mol/mL

**5**	+	4.19 *μ*mol/mL	+	2.09 *μ*mol/mL

**6**	2.08 *μ*mol/mL	2.08 *μ*mol/mL	4.16 *μ*mol/mL	0.13 *μ*mol/mL

**7**	+	3.96 *μ*mol/mL	+	1.98 *μ*mol/mL

**8**	3.10 *μ*mol/mL	3.10 *μ*mol/mL	3.10 *μ*mol/mL	1.55 *μ*mol/mL

**9**	+	+	+	0.40 *μ*mol/mL

**10**	+	+	+	0.41 *μ*mol/mL

**11**	1.58 *μ*mol/mL	1.58 *μ*mol/mL	0.78 *μ*mol/mL	0.024 *μ*mol/mL

**12**	2.85 *μ*mol/mL	2.85 *μ*mol/mL	2.85 *μ*mol/mL	1.42 *μ*mol/mL

Control of the environment	-	-	-	-

Nystatin	0.00043 *μ*mol/mL	0.00043 *μ*mol/mL	0.00043 *μ*mol/mL	0.00043*μ*mol/mL

Control of the microorganism	+	+	+	+

(+) indicates growth of the microorganism; (-) no growth microorganism.

**Table 2:** MFC values (*μ*mol/mL) and MFC/MIC ratio for compounds **1-12**.

Compounds	*Candida albicans*		*Candida glabrata*		*Candida krusei*		*Candida guilliermondi*	
	(ATCC 90028)		(ATCC 90030)		(ATCC 34135)		(ATCC 22017)	
	MFC	MFC/MIC	MFC	MFC/MIC	MFC	MFC/MIC	MFC	MFC/MIC
**1**	10.18	2	5.09	1	+		1.27	1

**2**	4.75	1	4.75	1	4.75	1	1.19	1

**3**	+		+		+		2.22	1

**4**	+		+		+		2.22	1

**5**	+		4.19	1	+		2.09	1

**6**	2.08	1	2.08	1	4.16	1	0.13	1

**7**	+		3.96	1	+		+	

**8**	3.10	1	3.10	1	3.10	1	1.55	2

**9**	+		+		+		0.40	1

**10**	+		+		+		0.41	1

**11**	1.58	1	1.58	1	0.78	1	0.024	1

**12**	2.85	1	2.85	1	2.85	1	+	

(+) The compounds present no MFC.

MFC/MIC relation ≥ 4: fungistatic activity. MFC/MIC < 4: fungicidal activity [14].

*∗* MFC: minimal fungicidal concentration; MIC: minimum inhibitory concentration.

**Table 3:** Minimum inhibitory concentration MIC (*μ*mol/mL) results for esters **1**-**12** against bacteria, Microdilution Technique.

Compounds	*Pseudomonas aeruginosa*	*Pseudomonas aeruginosa*	*Staphylococcus aureus*	*Staphylococcus aureus*
	ATCC 8027	102	ATCC 25925	47
**1**	+	+	5.09 *μ*mol/mL	+

**2**	+	+	+	+

**3**	+	+	+	+

**4**	+	+	+	+

**5**	+	+	+	+

**6**	+	+	+	+

**7**	+	+	+	+

**8**	+	+	+	+

**9**	+	+	+	+

**10**	+	+	+	+

**11**	+	+	+	+

**12**	+	+	+	+

Control of the environment	-	-	-	-

Chloramphenicol	0.3095 *μ*mol/mL	0.3095 *μ*mol/mL	0.3095 *μ*mol/mL	0.3095 *μ*mol/mL

Control of the microorganism	+	+	+	+

(+) indicates growth of the microorganism; (-) no microorganism growth.

**Table 4:** Compounds **1**-**12 **with docking score, interaction data, and distance between the protein residues.

Compounds	PLANT Score	M. Dock Score	Rerank Score	Amino acid	Group involved	Distance (Å)
**1**	-76.5806	-92.0557	-65.2081	H_2_O175 (O)	CO(O)	3.11

**2**	-71.2322	-98.2911	-32.276	Thr 260 (O)	CO(O)	3.22
				H_2_O87 (O)	CO(O)	2.66

**3**	-78.216	-93.7199	-71.2272	H_2_O87 (O)	CO(O)	3.01

**4**	-73.6695	-95.7275	-61.0177	H_2_O87 (O)	CO(O)	3.08
				Thr 260 (O)	CO(O)	3.07

**5**	-82.0128	-105.117	-76.2732	H_2_O87 (O)	CO(O)	3.00
				Thr 260 (O)	CO(O)	2.98

**6**	-77.3574	-93.9184	-71.3815	H_2_O175 (O)	CO(O)	2.76
				H_2_O87 (O)	O-2	2.87

**7**	-83.0449	-118.622	-86.715	H_2_O87 (O)	CO(O)	2.96

**8**	-92.9589	-140.652	-102.107	Thr 260 (O)	CO(O)	2.97
				H_2_O87 (O)	CO(O)	3.00
				H_2_O87 (O)	O	3.34

**9**	-84.0241	-108.849	-84.857	H_2_O175 (O)	CO(O)	2.82

**10**	-77.7839	-113.068	-59.4905	Gln 72 (N)	CO(O)	2.69
				H_2_O175 (O)	CO(O)	3.12
				H_2_O175 (O)	O-1	3.31
				H_2_O87 (O)	O-2	3.52

**11**	-85.142	-115.206	-85.2394	Gln 72 (N)	CO(O)	2.98
				H_2_O175 (O)	O	3.09

**12**	-81.6806	-131.112	-91.5155	H_2_O175 (O)	O	3.59

## Data Availability

The article data used to support the findings of this study titled “Antimicrobial Activity of 4-Chlorocinnamic Acid Derivatives” have been deposited in the Federal University of Paraíba repository https://repositorio.ufpb.br/jspui/handle/123456789/13687.
